# Coefficient Estimates for Initial Taylor-Maclaurin Coefficients for a Subclass of Analytic and Bi-Univalent Functions Defined by Al-Oboudi Differential Operator

**DOI:** 10.1155/2013/171039

**Published:** 2013-12-29

**Authors:** Serap Bulut

**Affiliations:** Civil Aviation College, Kocaeli University, Arslanbey Campus, 41285 İzmit-Kocaeli, Turkey

## Abstract

We introduce and investigate an interesting subclass *𝒩𝒫*_Σ_^*λ*,*δ*^(*n*, *β*; *h*) of analytic and bi-univalent functions in the open unit disk *𝕌*. For functions belonging to the class *𝒩𝒫*_Σ_^*λ*,*δ*^(*n*, *β*; *h*), we obtain estimates on the first two Taylor-Maclaurin coefficients *|a*_2_ *|* and *|a*_3_ *|*.

## 1. Introduction

Let ℝ = (−*∞*, *∞*) be the set of real numbers, *ℂ* the set of complex numbers, and

(1) $$\begin{array}{c} \mathbb{N}:=\left\{1,2,3,\ldots \right\}={\mathbb{N}}_{0}\setminus \left\{0\right\} \end{array}$$

the set of positive integers.

Let *𝒜* denote the class of all functions of the form

(2) $$\begin{array}{c} f\left(z\right)=z+\sum_{k=2}^{\infty }a_{k}z^{k} \end{array}$$

which are analytic in the open unit disk

(3) $$\begin{array}{c} 𝕌=\left\{z:z\in \mathbb{C}\;\;\text{and}\;\left|z\right|<1\right\}. \end{array}$$

We also denote by *𝒮* the class of all functions in the normalized analytic function class *𝒜* which are univalent in *𝕌*.

For two functions *f* and *g*, analytic in *𝕌*, we say that the function *f* is subordinate to *g* in *𝕌* and write

(4) $$\begin{array}{c} f\left(z\right)≺g\left(z\right)\;\left(z\in 𝕌\right), \end{array}$$

if there exists a Schwarz function *ω*, which is analytic in *𝕌* with

(5) $$\begin{array}{c} \omega \left(0\right)=0,\;\;\left|\omega \left(z\right)\right|<1,\;\left(z\in 𝕌\right) \end{array}$$

such that

(6) $$\begin{array}{c} f\left(z\right)=g\left(\omega \left(z\right)\right),\;\left(z\in 𝕌\right). \end{array}$$

Indeed, it is known that

(7) $$\begin{array}{c} f\left(z\right)≺g\left(z\right),\;\left(z\in 𝕌\right)\Rightarrow f\left(0\right)=g\left(0\right),\\ f\left(𝕌\right)\subset g\left(𝕌\right). \end{array}$$

Furthermore, if the function *g* is univalent in *𝕌*, then we have the following equivalence:

(8) $$\begin{array}{c} f\left(z\right)≺g\left(z\right),\;\left(z\in 𝕌\right)\Leftrightarrow f\left(0\right)=g\left(0\right),\\ f\left(𝕌\right)\subset g\left(𝕌\right). \end{array}$$

For *f* ∈ *𝒜*, Al-Oboudi [1] introduced the following operator:

(9) $$\begin{array}{c} D_{\delta }^{0}f\left(z\right)=f\left(z\right), \end{array}$$

(10) $$\begin{array}{c} D_{\delta }^{1}f\left(z\right)=\left(1-\delta \right)f\left(z\right)+\delta zf^{\prime }\left(z\right)=:D_{\delta }f\left(z\right),\;\left(\delta \geq 0\right), \end{array}$$

(11) $$\begin{array}{c} D_{\delta }^{n}f\left(z\right)=D_{\delta }\left(D_{\delta }^{n-1}f\left(z\right)\right),\;\left(n\in \mathbb{N}\right). \end{array}$$

If *f* is given by (2), then from (10) and (11) we see that

(12) $$\begin{array}{c} D_{\delta }^{n}f\left(z\right)=z+\sum_{k=2}^{\infty }{\left[1+(k-1)\delta \right]}^{n}a_{k}z^{k},\;\left(n\in {\mathbb{N}}_{0}\right), \end{array}$$

with *D*_*δ*_^*n*^ *f*(0) = 0. When *δ* = 1, we get Sǎlǎgean's differential operator *D*_1_^*n*^ = *D* ^*n*^, [2].

Since univalent functions are one-to-one, they are invertible and the inverse functions need not be defined on the entire unit disk *𝕌*. In fact, the Koebe one-quarter theorem [3] ensures that the image of *𝕌* under every univalent function *f* ∈ *𝒮* contains a disk of radius 1/4. Thus every function *f* ∈ *𝒜* has an inverse *f* ^−1^, which is defined by

(13) $$\begin{array}{c} f^{-1}\left(f\left(z\right)\right)=z\;\left(z\in 𝕌\right),\\ f\left(f^{-1}\left(w\right)\right)=w\;\;\left(\left|w\right|<r_{0}\left(f\right);\;r_{0}\left(f\right)\geq \frac{1}{4}\right). \end{array}$$

In fact, the inverse function *f* ^−1^ is given by

(14) f−1(w)=w−a2w2+(2a22−a3)w3 −(5a23−5a2a3+a4)w4+⋯.

A function *f* ∈ *𝒜* is said to be bi-univalent in *𝕌* if both *f* and *f* ^−1^ are univalent in *𝕌*. Let Σ denote the class of bi-univalent functions in *𝕌* given by (2). For a brief history and interesting examples of functions in the class Σ, see [4] (see also [5, 6]). In fact, the aforecited work of Srivastava et al. [4] essentially revived the investigation of various subclasses of the bi-univalent function class Σ in recent years; it was followed by such works as those by Frasin and Aouf [7], Porwal and Darus [8], and others (see, e.g., [9–17]).

Motivated by the abovementioned works, we define the following subclass of function class Σ.

**Definition 1**

Let *h* : *𝕌* → *ℂ* be a convex univalent function such that

(15) $$\begin{array}{c} h\left(0\right)=1,\;\;h\left(\bar{z}\right)=\bar{h\left(z\right)}\;\left(z\in 𝕌;ℜ\left(h\left(z\right)\right)>0\right). \end{array}$$

A function *f*, defined by (2), is said to be in the class *𝒩𝒫*_Σ_^*λ*,*δ*^(*n*, *β*; *h*) if the following conditions are satisfied:

(16) f∈Σ,eiβ((1−λ)Dδnf(z)z+λ(Dδnf(z))′)≺h(z)cos⁡β+isinβ(z∈𝕌),eiβ((1−λ)Dδng(w)w+λ(Dδng(w))′)  ≺h(w)cos⁡β+i sinβ (w∈𝕌),

where *β* ∈ (−*π*/2, *π*/2), *λ* ≥ 1, the function *g* is given by

(17) g(w)=w−a2w2+(2a22−a3)w3 −(5a23−5a2a3+a4)w4+⋯,

and *D*_*δ*_^*n*^ is the Al-Oboudi differential operator.

**Remark 2**

If we set

(18) $$\begin{array}{c} h\left(z\right)=\frac{1+Az}{1+Bz}\;\left(-1\leq B<A\leq 1\right) \end{array}$$

in Definition 1, then the class *𝒩𝒫*_Σ_^*λ*,*δ*^(*n*, *β*; *h*) reduces to the class denoted by *𝒩𝒫*_Σ_^*λ*,*δ*^(*n*, *β*; *A*, *B*) which is the subclass of the functions *f* ∈ Σ satisfying

(19) eiβ((1−λ)Dδnf(z)z+λ(Dδnf(z))′)  ≺1+Az1+Bzcos⁡β+isinβ (z∈𝕌),eiβ((1−λ)Dδng(w)w+λ(Dδng(w))′)  ≺1+Aw1+Bwcos⁡β+i sinβ (w∈𝕌),

where *β* ∈ (−*π*/2, *π*/2), *λ* ≥ 1, the function *g* is defined by (17), and *D*_*δ*_^*n*^ is the Al-Oboudi differential operator.

**Remark 3**

If we set

(20) $$\begin{array}{c} h\left(z\right)=\frac{1+\left(1-2\alpha \right)z}{1-z}\;\left(0\leq \alpha <1\right) \end{array}$$

in Definition 1, then the class *𝒩𝒫*_Σ_^*λ*,*δ*^(*n*, *β*; *h*) reduces to the class denoted by *𝒩𝒫*_Σ_^*λ*,*δ*^(*n*, *β*, *α*) which is the subclass of the functions *f* ∈ Σ satisfying

(21) ℜ{eiβ((1−λ)Dδnf(z)z+λ(Dδnf(z))′)}>αcos⁡β(z∈𝕌),ℜ{eiβ((1−λ)Dδng(w)w+λ(Dδng(w))′)}>αcos⁡β(w∈𝕌),

where *β* ∈ (−*π*/2, *π*/2), *λ* ≥ 1, the function *g* is defined by (17), and *D*_*δ*_^*n*^ is the Al-Oboudi differential operator.

**Remark 4**

If we set

(22) $$\begin{array}{c} \delta =1,\;\;h\left(z\right)=\frac{1+\left(1-2\alpha \right)z}{1-z}\;\left(0\leq \alpha <1\right) \end{array}$$

in Definition 1, then the class *𝒩𝒫*_Σ_^*λ*,*δ*^(*n*, *β*; *h*) reduces to the class denoted by *𝒩𝒫*_Σ_^*λ*^(*n*, *β*, *α*) which is the subclass of the functions *f* ∈ Σ satisfying

(23) ℜ{eiβ((1−λ)Dnf(z)z+λ(Dnf(z))′)}>αcos⁡β(z∈𝕌),ℜ{eiβ((1−λ)Dng(w)w+λ(Dng(w))′)}>αcos⁡β(w∈𝕌),

where *β* ∈ (−*π*/2, *π*/2), *λ* ≥ 1, the function *g* is defined by (17), and *D* ^*n*^ is the Sǎlǎgean differential operator.

**Remark 5**

If we set

(24) $$\begin{array}{c} n=0,\;\;h\left(z\right)=\frac{1+\left(1-2\alpha \right)z}{1-z}\;\left(0\leq \alpha <1\right) \end{array}$$

in Definition 1, then the class *𝒩𝒫*_Σ_^*λ*,*δ*^(*n*, *β*; *h*) reduces to the class denoted by *𝒩𝒫*_Σ_^*λ*^(*β*, *α*) which is the subclass of the functions *f* ∈ Σ satisfying

(25) $$\begin{array}{c} ℜ\left\{e^{i\beta }\left(\left(1-\lambda \right)\frac{f\left(z\right)}{z}+\lambda f^{\prime }\left(z\right)\right)\right\}>\alpha \cos\beta \;\left(z\in 𝕌\right),\\ ℜ\left\{e^{i\beta }\left(\left(1-\lambda \right)\frac{g\left(w\right)}{w}+\lambda g^{\prime }\left(w\right)\right)\right\}>\alpha \cos\beta \;\left(w\in 𝕌\right), \end{array}$$

where *β* ∈ (−*π*/2, *π*/2), *λ* ≥ 1, and the function *g* is defined by (17).

**Remark 6**

If we set

(26) $$\begin{array}{c} n=0,\;\;\lambda =1,\;\;h\left(z\right)=\frac{1+\left(1-2\alpha \right)z}{1-z}\;\left(0\leq \alpha <1\right) \end{array}$$

in Definition 1, then the class *𝒩𝒫*_Σ_^*λ*,*δ*^(*n*, *β*; *h*) reduces to the class denoted by *𝒩𝒫*_Σ_(*β*, *α*) which is the subclass of the functions *f* ∈ Σ satisfying

(27) $$\begin{array}{c} ℜ\left\{e^{i\beta }f^{\prime }\left(z\right)\right\}>\alpha \cos\beta \;\left(z\in 𝕌\right),\\ ℜ\left\{e^{i\beta }g^{\prime }\left(w\right)\right\}>\alpha \cos\beta \;\left(w\in 𝕌\right), \end{array}$$

where *β* ∈ (−*π*/2, *π*/2) and the function *g* is defined by (17).

We note that

(28) $$\begin{array}{c} 𝒩{𝒫}_{\Sigma }^{\lambda }\left(n,0,\alpha \right)={ℋ}_{\Sigma }\left(n,\alpha ,\lambda \right)\;\left(\text{see}\;\left[8\right]\right),\\ 𝒩{𝒫}_{\Sigma }^{\lambda }\left(0,\alpha \right)={ℬ}_{\Sigma }\left(\alpha ,\lambda \right)\;\left(\text{see}\;\left[7\right]\right),\\ 𝒩{𝒫}_{\Sigma }\left(0,\alpha \right)={ℋ}_{\Sigma }\left(\alpha \right)\;\left(\text{see}\;\left[4\right]\right). \end{array}$$

Firstly, in order to derive our main results, we need the following lemma.

**Lemma 7 (see [18])**

Let the function *h*(*z*) given by

(29) $$\begin{array}{c} h\left(z\right)=\sum_{n=1}^{\infty }B_{n}z^{n} \end{array}$$

be convex in *𝕌*. Suppose also that the function *φ*(*z*) given by

(30) $$\begin{array}{c} \varphi \left(z\right)=\sum_{n=1}^{\infty }c_{n}z^{n} \end{array}$$

is holomorphic in *𝕌*. If *φ*(*z*)≺*h*(*z*)  (*z* ∈ *𝕌*), then

(31) $$\begin{array}{c} \left|c_{n}\right|\leq \left|B_{1}\right|\;\left(n\in \mathbb{N}\right). \end{array}$$

The object of the present paper is to find estimates on the Taylor-Maclaurin coefficients |*a*_2_| and |*a*_3_| for functions in this new subclass *𝒩𝒫*_Σ_^*λ*,*δ*^(*n*, *β*; *h*) of the function class Σ.

## 2. A Set of General Coefficient Estimates

In this section, we state and prove our general results involving the bi-univalent function class *𝒩𝒫*_Σ_^*λ*,*δ*^(*n*, *β*; *h*) given by Definition 1.

**Theorem 8**

Let the function *f*(*z*) given by the Taylor-Maclaurin series expansion (2) be in the function class

(32) $$\begin{array}{c} 𝒩{𝒫}_{\Sigma }^{\lambda ,\delta }\left(n,\beta ;h\right)\;\left(\beta \in \left(-\pi /2,\pi /2\right),\;\lambda \geq 1,\;\delta \geq 0,\;n\in {\mathbb{N}}_{0}\right) \end{array}$$

with

(33) $$\begin{array}{c} h\left(z\right)=1+B_{1}z+B_{2}z^{2}+\cdots . \end{array}$$

Then

(34) $$\begin{array}{c} \left|a_{2}\right|\leq \min\left\{\frac{\left|B_{1}\right|\cos\beta }{{\left(1+\delta \right)}^{n}\left(1+\lambda \right)},\sqrt{\frac{\left|B_{1}\right|\cos\beta }{{\left(1+2\delta \right)}^{n}\left(1+2\lambda \right)}}\right\}, \end{array}$$

(35) $$\begin{array}{c} \left|a_{3}\right|\leq \frac{\left|B_{1}\right|\cos\beta }{{\left(1+2\delta \right)}^{n}\left(1+2\lambda \right)}. \end{array}$$

**Proof**

It follows from (16) that

(36) eiβ((1−λ)Dδnf(z)z+λ(Dδnf(z))′)  =p(z)cos⁡β+isinβ (z∈𝕌),

(37) eiβ((1−λ)Dδng(w)w+λ(Dδng(w))′)  =q(w)cos⁡β+isinβ (w∈𝕌),

where *p*(*z*)≺*h*(*z*) and *q*(*w*)≺*h*(*w*) have the following Taylor-Maclaurin series expansions:

(38) $$\begin{array}{c} p\left(z\right)=1+p_{1}z+p_{2}z^{2}+\cdots , \end{array}$$

(39) $$\begin{array}{c} q\left(w\right)=1+q_{1}w+q_{2}w^{2}+\cdots , \end{array}$$

respectively. Now, upon equating the coefficients in (36) and (37), we get

(40) $$\begin{array}{c} e^{i\beta }{\left(1+\delta \right)}^{n}\left(1+\lambda \right)a_{2}=p_{1}\cos\beta , \end{array}$$

(41) $$\begin{array}{c} e^{i\beta }{\left(1+2\delta \right)}^{n}\left(1+2\lambda \right)a_{3}=p_{2}\cos\beta , \end{array}$$

(42) $$\begin{array}{c} -e^{i\beta }{\left(1+\delta \right)}^{n}\left(1+\lambda \right)a_{2}=q_{1}\cos\beta , \end{array}$$

(43) eiβ[−(1+2δ)n(1+2λ)a3+2(1+2δ)n(1+2λ)a22]  =q2cos⁡β.

From (40) and (42), we obtain

(44) $$\begin{array}{c} p_{1}=-q_{1}, \end{array}$$

(45) $$\begin{array}{c} 2e^{2i\beta }{\left(1+\delta \right)}^{2n}{\left(1+\lambda \right)}^{2}a_{2}^{2}=\left(p_{1}^{2}+q_{1}^{2}\right)\cos^{2}\beta . \end{array}$$

Also, from (41) and (43), we find that

(46) $$\begin{array}{c} a_{2}^{2}=\frac{e^{-i\beta }\left(p_{2}+q_{2}\right)\cos\beta }{2{\left(1+2\delta \right)}^{n}\left(1+2\lambda \right)}. \end{array}$$

Since *p*, *q* ∈ *h*(*𝕌*), according to Lemma 7, we immediately have

(47) $$\begin{array}{c} \left|p_{k}\right|=\left|\frac{p^{\left(k\right)}\left(0\right)}{k!}\right|\leq \left|B_{1}\right|\;\left(k\in \mathbb{N}\right),\\ \left|q_{k}\right|=\left|\frac{q^{\left(k\right)}\left(0\right)}{k!}\right|\leq \left|B_{1}\right|\;\left(k\in \mathbb{N}\right). \end{array}$$

Applying (47) and Lemma 7 for the coefficients *p*_1_, *p*_2_, *q*_1_, and *q*_2_, from the equalities (45) and (46), we obtain

(48) $$\begin{array}{c} {\left|a_{2}\right|}^{2}\leq \frac{{\left|B_{1}\right|}^{2}\cos^{2}\beta }{{\left(1+\delta \right)}^{2n}{\left(1+\lambda \right)}^{2}}, \end{array}$$

(49) $$\begin{array}{c} {\left|a_{2}\right|}^{2}\leq \frac{\left|B_{1}\right|\cos\beta }{{\left(1+2\delta \right)}^{n}\left(1+2\lambda \right)}, \end{array}$$

respectively. So we get the desired estimate on the coefficient |*a*_2_| as asserted in (34).

Next, in order to find the bound on the coefficient |*a*_3_|, we subtract (43) from (41). We thus get

(50) 2(1+2δ)n(1+2λ)a3−2(1+2δ)n(1+2λ)a22  =e−iβ(p2−q2)cos⁡β.

Upon substituting the value of *a*_2_^2^ from (45) into (50), it follows that

(51) $$\begin{array}{c} a_{3}=\frac{e^{-2i\beta }\left(p_{1}^{2}+q_{1}^{2}\right)\cos^{2}\beta }{2{\left(1+\delta \right)}^{2n}{\left(1+\lambda \right)}^{2}}+\frac{e^{-i\beta }\left(p_{2}-q_{2}\right)\cos\beta }{2{\left(1+2\delta \right)}^{n}\left(1+2\lambda \right)}. \end{array}$$

So we get

(52) $$\begin{array}{c} \left|a_{3}\right|\leq \frac{{\left|B_{1}\right|}^{2}\cos^{2}\beta }{{\left(1+\delta \right)}^{2n}{\left(1+\lambda \right)}^{2}}+\frac{\left|B_{1}\right|\cos\beta }{{\left(1+2\delta \right)}^{n}\left(1+2\lambda \right)}. \end{array}$$

On the other hand, upon substituting the value of *a*_2_^2^ from (46) into (50), it follows that

(53) $$\begin{array}{c} a_{3}=\frac{e^{-i\beta }\left(p_{2}+q_{2}\right)\cos\beta }{2{\left(1+2\delta \right)}^{n}\left(1+2\lambda \right)}+\frac{e^{-i\beta }\left(p_{2}-q_{2}\right)\cos\beta }{2{\left(1+2\delta \right)}^{n}\left(1+2\lambda \right)}. \end{array}$$

And we get

(54) $$\begin{array}{c} \left|a_{3}\right|\leq \frac{\left|B_{1}\right|\cos\beta }{{\left(1+2\delta \right)}^{n}\left(1+2\lambda \right)}. \end{array}$$

Comparing the inequalities in (52) and (54) completes the proof of Theorem 8.

## 3. Corollaries and Consequences

By setting

(55) $$\begin{array}{c} h\left(z\right)=\frac{1+Az}{1+Bz}\;\left(-1\leq B<A\leq 1\right) \end{array}$$

in Theorem 8, we have the following corollary.

**Corollary 9**

Let the function *f*(*z*) given by the Taylor-Maclaurin series expansion (2) be in the function class

(56) 𝒩𝒫Σλ,δ(n,β;A,B)(β∈(−π/2,π/2),λ≥1,δ≥0,−1≤B<A≤1,n∈ℕ0).

Then

(57) $$\begin{array}{c} \left|a_{2}\right|\leq \min\left\{\frac{\left(A-B\right)\cos\beta }{{\left(1+\delta \right)}^{n}\left(1+\lambda \right)},\sqrt{\frac{\left(A-B\right)\cos\beta }{{\left(1+2\delta \right)}^{n}\left(1+2\lambda \right)}}\right\},\\ \left|a_{3}\right|\leq \frac{\left(A-B\right)\cos\beta }{{\left(1+2\delta \right)}^{n}\left(1+2\lambda \right)}. \end{array}$$

By setting

(58) $$\begin{array}{c} h\left(z\right)=\frac{1+\left(1-2\alpha \right)z}{1-z}\;\left(0\leq \alpha <1\right) \end{array}$$

in Theorem 8, we have the following corollary.

**Corollary 10**

Let the function *f*(*z*) given by the Taylor-Maclaurin series expansion (2) be in the function class

(59) 𝒩𝒫Σλ,δ(n,β,α)(β∈(−π/2,π/2),λ≥1,δ≥0,0≤α<1,n∈ℕ0).

Then

(60) $$\begin{array}{c} \left|a_{2}\right|\leq \min\left\{\frac{2\left(1-\alpha \right)\cos\beta }{{\left(1+\delta \right)}^{n}\left(1+\lambda \right)},\sqrt{\frac{2\left(1-\alpha \right)\cos\beta }{{\left(1+2\delta \right)}^{n}\left(1+2\lambda \right)}}\right\},\\ \left|a_{3}\right|\leq \frac{2\left(1-\alpha \right)\cos\beta }{{\left(1+2\delta \right)}^{n}\left(1+2\lambda \right)}. \end{array}$$

By setting

(61) $$\begin{array}{c} \delta =1,\;\;h\left(z\right)=\frac{1+\left(1-2\alpha \right)z}{1-z}\;\left(0\leq \alpha <1\right) \end{array}$$

in Theorem 8, we have the following corollary.

**Corollary 11**

Let the function *f*(*z*) given by the Taylor-Maclaurin series expansion (2) be in the function class

(62) 𝒩𝒫Σλ(n,β,α)(β∈(−π/2,π/2),λ≥1,0≤α<1,n∈ℕ0).

Then

(63) $$\begin{array}{c} \left|a_{2}\right|\leq \min\left\{\frac{2\left(1-\alpha \right)\cos\beta }{2^{n}\left(1+\lambda \right)},\sqrt{\frac{2\left(1-\alpha \right)\cos\beta }{3^{n}\left(1+2\lambda \right)}}\right\},\\ \left|a_{3}\right|\leq \frac{2\left(1-\alpha \right)\cos\beta }{3^{n}\left(1+2\lambda \right)}. \end{array}$$

**Remark 12**

When *β* = 0, Corollary 11 is an improvement of the following estimates obtained by Porwal and Darus [8].

**Corollary 13 (see [8])**

Let the function *f*(*z*) given by the Taylor-Maclaurin series expansion (2) be in the function class

(64) $$\begin{array}{c} {ℋ}_{\Sigma }\left(n,\alpha ,\lambda \right)\;\left(\lambda \geq 1,\;\;0\leq \alpha <1,\;\;n\in {\mathbb{N}}_{0}\right). \end{array}$$

Then

(65) $$\begin{array}{c} \left|a_{2}\right|\leq \sqrt{\frac{2\left(1-\alpha \right)}{3^{n}\left(1+2\lambda \right)}},\\ \left|a_{3}\right|\leq \frac{4{\left(1-\alpha \right)}^{2}}{2^{2n}{\left(1+\lambda \right)}^{2}}+\frac{2\left(1-\alpha \right)}{3^{n}\left(1+2\lambda \right)}. \end{array}$$

By setting

(66) $$\begin{array}{c} n=0,\;\;h\left(z\right)=\frac{1+\left(1-2\alpha \right)z}{1-z}\;\left(0\leq \alpha <1\right) \end{array}$$

in Theorem 8, we have the following corollary.

**Corollary 14**

Let the function *f*(*z*) given by the Taylor-Maclaurin series expansion (2) be in the function class

(67) $$\begin{array}{c} 𝒩{𝒫}_{\Sigma }^{\lambda }\left(\beta ,\alpha \right)\;\left(\beta \in \left(-\pi /2,\pi /2\right),\;\lambda \geq 1,\;0\leq \alpha <1\right). \end{array}$$

Then

(68) $$\begin{array}{c} \left|a_{2}\right|\leq \min\left\{\frac{2\left(1-\alpha \right)\cos\beta }{1+\lambda },\sqrt{\frac{2\left(1-\alpha \right)\cos\beta }{1+2\lambda }}\right\},\\ \left|a_{3}\right|\leq \frac{2\left(1-\alpha \right)\cos\beta }{1+2\lambda }. \end{array}$$

**Remark 15**

When *β* = 0, Corollary 14 is an improvement of the following estimates obtained by Frasin and Aouf [7].

**Corollary 16 (see [7])**

Let the function *f*(*z*) given by the Taylor-Maclaurin series expansion (2) be in the function class

(69) $$\begin{array}{c} {ℬ}_{\Sigma }\left(\alpha ,\lambda \right)\;\left(\lambda \geq 1,\;0\leq \alpha <1\right). \end{array}$$

Then

(70) $$\begin{array}{c} \left|a_{2}\right|\leq \sqrt{\frac{2\left(1-\alpha \right)}{1+2\lambda }},\\ \left|a_{3}\right|\leq \frac{4{\left(1-\alpha \right)}^{2}}{{\left(1+\lambda \right)}^{2}}+\frac{2\left(1-\alpha \right)}{1+2\lambda }. \end{array}$$

By setting

(71) $$\begin{array}{c} n=0,\;\lambda =1,\;h\left(z\right)=\frac{1+\left(1-2\alpha \right)z}{1-z}\;\left(0\leq \alpha <1\right) \end{array}$$

in Theorem 8, we have the following corollary.

**Corollary 17**

Let the function *f*(*z*) given by the Taylor-Maclaurin series expansion (2) be in the function class

(72) $$\begin{array}{c} 𝒩{𝒫}_{\Sigma }\left(\beta ,\alpha \right)\;\left(\beta \in \left(-\pi /2,\pi /2\right),0\leq \alpha <1\right). \end{array}$$

Then

(73) $$\begin{array}{c} \left|a_{2}\right|\leq \min\left\{\left(1-\alpha \right)\cos\beta ,\sqrt{\frac{2\left(1-\alpha \right)\cos\beta }{3}}\right\},\\ \left|a_{3}\right|\leq \frac{2\left(1-\alpha \right)\cos\beta }{3}. \end{array}$$

**Remark 18**

When *β* = 0, Corollary 17 is an improvement of the following estimates obtained by Srivastava et al. [4].

**Corollary 19 (see [4])**

Let the function *f*(*z*) given by the Taylor-Maclaurin series expansion (2) be in the function class

(74) $$\begin{array}{c} {ℋ}_{\Sigma }\left(\alpha \right)\;\left(0\leq \alpha <1\right). \end{array}$$

Then

(75) $$\begin{array}{c} \left|a_{2}\right|\leq \sqrt{\frac{2\left(1-\alpha \right)}{3},}\\ \left|a_{3}\right|\leq \frac{\left(1-\alpha \right)\left(5-3\alpha \right)}{3}. \end{array}$$

## References

[1] Al-Oboudi FM (2004). On univalent functions defined by a generalized Sălăgean operator. *International Journal of Mathematics and Mathematical Sciences*.

[2] Sǎlǎgean GS (1983). Subclasses of univalent functions. *Complex Analysis-Fifth Romanian-Finnish Seminar, Part 1 (Bucharest, 1981)*.

[3] Duren PL (1983). *Univalent Functions*.

[4] Srivastava HM, Mishra AK, Gochhayat P (2010). Certain subclasses of analytic and bi-univalent functions. *Applied Mathematics Letters*.

[5] Brannan DA, Taha TS, Mazhar SM, Hamoui A, Faour NS (1988). On some classes of bi-univalent functions. *Mathematical Analysis and Its Applications*.

[6] Brannan DA, Taha TS (1986). On some classes of bi-univalent functions. *Studia Universitatis Babeş-Bolyai Mathematica*.

[7] Frasin BA, Aouf MK (2011). New subclasses of bi-univalent functions. *Applied Mathematics Letters*.

[8] Porwal S, Darus M (2013). On a new subclass of bi-univalent functions. *Journal of the Egyptian Mathematical Society*.

[9] Bulut S (2013). Coefficient estimates for a class of analytic and bi-univalent functions. *Novi Sad Journal of Mathematics*.

[10] Bulut S (2013). Coefficient estimates for new subclasses of analytic and bi-univalent functions defined by Al-Oboudi differential operator. *Journal of Function Spaces and Applications*.

[11] Çağlar M, Orhan H, Yağmur N (2013). Coefficient bounds for new subclasses of bi-univalent functions. *Filomat*.

[12] Goyal SP, Goswami P (2012). Estimate for initial Maclaurin coefficients of bi-univalent functions for a class defined by fractional derivatives. *Journal of the Egyptian Mathematical Society*.

[13] Hayami T, Owa S (2012). Coefficient bounds for bi-univalent functions. *Pan-American Mathematical Journal*.

[14] Orhan H, Magesh N, Balaji VK Initial coefficient bounds for a general class of bi-univalent functions.

[15] Srivastava HM, Bulut S, Çağlar M, Yağmur N (2013). Coefficient estimates for a general subclass of analytic and bi-univalent functions. *Filomat*.

[16] Xu QH, Gui YC, Srivastava HM (2012). Coefficient estimates for a certain subclass of analytic and bi-univalent functions. *Applied Mathematics Letters*.

[17] Xu QH, Xiao HG, Srivastava HM (2012). A certain general subclass of analytic and bi-univalent functions and associated coefficient estimate problems. *Applied Mathematics and Computation*.

[18] Rogosinski W (1943). On the coefficients of subordinate functions. *Proceedings of the London Mathematical Society*.

